# Development and Psychometric Evaluation of a Mental Health Help-Seeking Scale for Korean Adolescents

**DOI:** 10.3390/children13081108

**Published:** 2026-08-19

**Authors:** Jihye Shin, Kuem Sun Han

**Affiliations:** 1 College of Nursing, Catholic University of Pusan, Busan 46252, Republic of Korea; jhshin@cup.ac.kr; 2 College of Nursing, Korea University, Seoul 02841, Republic of Korea

**Keywords:** mental health, help-seeking behavior, adolescent, instrument development, psychometric validation

## Abstract

**Highlights:**

**What are the main findings?**

- A developmentally and culturally grounded 16-item scale developed from adolescents’ experiences yielded a theoretically interpretable three-factor structure with preliminary psychometric support in independent EFA and CFA samples.
- Unlike adult-oriented measures focused primarily on attitudes toward professional help, the scale proposes three dimensions: Mental Health Help-Seeking Willingness, Overcoming Stigma in Mental Health Help-Seeking, and Knowledge of Mental Health Help-Seeking Resources.

**What are the implications of the main findings?**

- Assessing these distinct dimensions may help clarify whether delayed help-seeking is related to limited willingness, stigma-related barriers, or insufficient knowledge of available support resources.
- The scale shows potential for informing and evaluating culturally responsive early-prevention initiatives designed to facilitate adolescents’ timely access to support in educational, clinical, and community settings.

**Abstract:**

**Background/Objectives:** Existing mental health help-seeking measures may not adequately reflect adolescents’ developmental and sociocultural contexts. This study developed and evaluated a mental health help-seeking scale for Korean adolescents. **Methods:** Following DeVellis’ guidelines, items were generated through concept analysis and in-depth interviews. Expert review reduced 50 initial items to 33 preliminary items. Data were collected from 500 adolescents in Gyeongsangbuk-do, Republic of Korea. EFA and CFA were conducted using separate samples of 250 participants. **Results:** The final scale comprised 16 items across three factors: Mental Health Help-Seeking Willingness, Overcoming Stigma in Mental Health Help-Seeking, and Knowledge of Mental Health Help-Seeking Resources. The factors explained 46.4% of the variance, and CFA provided preliminary support for the three-factor structure. Cronbach’s α was 0.85 overall and ranged from 0.73 to 0.85 across subscales. **Conclusions:** The scale provides preliminary evidence of reliability and validity for assessing mental health help-seeking among Korean adolescents and may be useful in research and early-prevention settings.

## 1. Introduction

Mental health problems among adolescents have become a major global public health concern. According to UNICEF, one in seven adolescents experiences a mental health problem [1]. In South Korea, the lifetime prevalence of mental disorders among children and adolescents has been reported as 16.1%, with a current prevalence of 7.1% [2]. Furthermore, the adolescent suicide rate in South Korea is among the highest among the Organisation for Economic Co-operation and Development (OECD) countries [3]. These findings highlight the importance of strengthening early prevention and intervention strategies to promote adolescent mental health. Despite the growing need for mental health care, many adolescents experiencing psychological distress do not receive appropriate support. In South Korea, the utilization rate of mental health services among adolescents has been reported to be only 4.3% [3], indicating a substantial gap between mental health needs and actual service utilization.

Mental health help-seeking is increasingly understood as a multidimensional process that begins with recognizing psychological distress, overcoming internal and external barriers, identifying appropriate sources of support, and ultimately seeking professional or informal assistance [4]. Timely help-seeking facilitates early identification and intervention, reduces the severity and progression of mental health problems [5], and helps prevent adverse mental health outcomes from persisting into adulthood [6]. However, adolescents often encounter barriers to seeking help, including stigma, negative attitudes toward mental illness, and limited mental health literacy [7,8]. Consequently, promoting mental health help-seeking has become an important component of adolescent mental health promotion and suicide prevention efforts.

In response to these challenges, South Korea has increasingly emphasized prevention-oriented mental health policies by strengthening school-based mental health promotion and suicide prevention programs and expanding access to community-based mental health services. Nevertheless, standardized instruments capable of evaluating the effectiveness of these initiatives and accurately assessing adolescents’ mental health help-seeking remain limited.

Although several instruments have been developed to assess mental health help-seeking, most were originally designed for adults or adapted from measures developed in other cultural settings, limiting their ability to adequately reflect adolescents’ developmental characteristics and sociocultural context [9,10,11,12]. In South Korea, previous studies have predominantly used modified or translated adult instruments [13,14,15], and few measures have been specifically developed to reflect the experiences and language of Korean adolescents. Furthermore, the concepts of help request, help-seeking, and service utilization have frequently been used interchangeably, resulting in conceptual ambiguity and inconsistency in measurement [16]. In this study, attitudes toward professional help refer to individuals’ evaluations of seeking professional support, whereas help-seeking readiness refers to the willingness and perceived capacity to seek help, including overcoming anticipated stigma and identifying available resources. Actual help-seeking behavior, in contrast, involves approaching or using a source of support and may additionally be influenced by contextual and access-related factors. The present scale was therefore developed to assess help-seeking readiness rather than actual service utilization.

Because help-seeking behaviors are influenced by developmental characteristics and sociocultural context, culturally appropriate and psychometrically sound instruments are essential for identifying adolescents who may require support, evaluating school- and community-based prevention programs, and informing evidence-based mental health promotion strategies. Therefore, this study aimed to develop and validate an instrument for assessing mental health help-seeking among Korean adolescents that reflects their developmental characteristics and sociocultural context. To achieve this aim, the conceptual dimensions of adolescent mental health help-seeking were identified through concept analysis, and the reliability and validity of the developed instrument were subsequently evaluated.

## 2. Materials and Methods

### 2.1. Study Design

This methodological study was conducted to develop and validate an instrument for assessing mental health help-seeking among Korean adolescents. Instrument development followed the eight-step scale development framework proposed by DeVellis [17], including concept clarification, item generation, scale format selection, content validity evaluation, pilot testing, psychometric evaluation, and instrument refinement. The completeness of reporting was reviewed with reference to relevant recommendations from the COSMIN Reporting Guideline. The completed COSMIN-informed checklist is provided in Supplementary Table S1.

### 2.2. Instrument Development

#### 2.2.1. Conceptualization of Mental Health Help-Seeking

The conceptual framework for the instrument was established through Rodgers’ evolutionary concept analysis [18], which combines evidence from the literature with empirical observations to clarify and redefine a concept. The concept analysis consisted of three integrated phases: a theoretical review, fieldwork, and final synthesis, through which adolescent mental health help-seeking was conceptualized as a multidimensional process rather than a single help-seeking behavior.

In the theoretical phase, the domestic and international literature on adolescent mental health help-seeking was systematically reviewed to identify the defining attributes, antecedents, and consequences of the concept. Literature searches were conducted using PubMed, Web of Science, CINAHL, PsycARTICLES, EMBASE, and the Korean database RISS. The search combined the terms mental health help-seeking, mental health help-seeking behavior, adolescent, and their Korean equivalents. Publications from 1991, when the concept first appeared in the literature, through 2025 were considered.

To complement the literature review, fieldwork was conducted through individual in-depth interviews with 10 adolescents using purposive sampling. Participants were recruited between April and May 2025. Semi-structured interviews explored adolescents’ experiences, perceptions, and decision-making processes related to seeking help for mental health concerns. Data collection and analysis were performed concurrently until no new conceptual information emerged.

Finally, findings from the literature review and fieldwork were compared and integrated to establish the conceptual framework of adolescent mental health help-seeking. Five core attributes were identified: recognizing psychological distress, overcoming perceived stigma, maintaining a positive attitude toward seeking help, identifying appropriate help resources, and making the decision to seek help. These attributes represented key determinants that precede and facilitate adolescent mental health help-seeking and served as the conceptual basis for item development (Table 1).

#### 2.2.2. Item Generation

Items were generated based on the conceptual framework established through concept analysis. An initial pool of 50 items was developed by integrating statements derived from the literature review and in-depth interviews with adolescents. The preliminary items were reviewed by the research team, including experts in nursing, counseling psychology, and psychiatric nursing, to evaluate conceptual relevance, clarity, and redundancy. Items with overlapping meanings were consolidated or removed, and wording was refined to improve clarity and age appropriateness while preserving the conceptual content. This process resulted in a preliminary pool of 50 items for content validity evaluation. Reverse-worded items were reverse-scored before item analysis and reliability testing (Supplementary Table S2).

#### 2.2.3. Scale Format Selection

Consistent with DeVellis’ recommendation that the response format should be determined during item development [17], a five-point Likert scale was selected because it is widely used to measure attitudes, beliefs, and behavioral intentions. Responses ranged from 1 (“strongly disagree”) to 5 (“strongly agree”), with higher scores indicating greater mental health help-seeking.

#### 2.2.4. Content Validity and Pilot Testing

Content validity was evaluated by a panel of eight experts consisting of one psychiatrist, one nursing professor, two psychiatric nurse practitioners, one mental health nurse, two elementary school teachers, and one middle school teacher. Each item was rated on a four-point relevance scale, and the Item-level Content Validity Index (I-CVI) and Scale-level Content Validity Index/Average (S-CVI/Ave) were calculated. Following the recommendation of Polit and Beck, items with an I-CVI below 0.78 were removed. In addition, items with overlapping content were eliminated, and wording was revised to improve clarity and comprehensibility for adolescents. The resulting preliminary instrument consisted of 33 items, with an S-CVI/Ave of 0.96.

Face validity was subsequently assessed through a pilot test involving 10 adolescents. Participants completed the questionnaire and provided feedback regarding item clarity, comprehension, and response burden. Completion required approximately 10–15 min. Based on participant feedback, explanatory wording was added to clarify the meaning of “mental health professionals,” whereas no items were added or removed. The 33-item preliminary instrument was therefore retained for psychometric evaluation. Reverse-worded items were reverse-scored before item analysis and reliability testing (Supplementary Table S3).

### 2.3. Psychometric Evaluation

#### 2.3.1. Participants

Participants were adolescents enrolled in elementary and middle schools in Gyeongbuk Province, South Korea. Eligible participants were students attending regular school classes who were able to communicate in Korean and who voluntarily agreed to participate. Written parental consent was obtained for all participants prior to data collection. Adolescents with language, developmental, or cognitive disorders that could interfere with questionnaire completion were excluded. Because the purpose of this study was to develop an instrument assessing adolescents’ readiness and propensity to seek help for mental health concerns rather than actual utilization of mental health services, eligibility was not restricted to adolescents with previous mental health help-seeking experience. This approach is consistent with the conceptualization of mental health help-seeking as a multidimensional construct encompassing willingness, perceived stigma, and knowledge of available help resources, all of which are relevant to adolescents regardless of prior service use. Separate samples were recruited for exploratory factor analysis (EFA) and confirmatory factor analysis (CFA), as using the same sample for both analyses may compromise the accuracy of construct validation [19]. Sample size was determined according to recommendations for factor analysis, which suggest recruiting at least 200 participants or five to ten participants per item when the instrument contains fewer than 40 items [20]. A minimum of 200 participants was required for each factor analysis. Allowing for an anticipated nonresponse or exclusion rate of approximately 25%, the target sample size was set at 250 participants for each analysis, resulting in a total target sample of 500.

#### 2.3.2. Data Collection

Data were collected between 8 July and 31 August 2025, from elementary and middle schools in Gyeongbuk Province, South Korea. Schools that agreed to participate were identified through convenience sampling, and eligible students were invited to participate after receiving approval from the school administration. Written informed consent was obtained from both the participants and their legal guardians before questionnaire administration. To ensure the independence of psychometric validation, participants for the EFA and CFA were recruited as two separate samples during different rounds of data collection, and no individual participated in both analyses. The questionnaires were administered by the researcher in classroom settings and required approximately 20 min to complete.

#### 2.3.3. Statistical Analysis

Psychometric evaluation was performed using IBM SPSS Statistics version 30.0 (IBM Corp., Armonk, NY, USA), IBM SPSS AMOS version 29.0 and Microsoft Excel.

Descriptive statistics were used to summarize participants’ characteristics and item distributions. Item analysis included the examination of item means, standard deviations, skewness, kurtosis, and corrected item–total correlations. Construct validity was evaluated through exploratory factor analysis (EFA) followed by confirmatory factor analysis (CFA) using independent samples. EFA was conducted using principal axis factoring with direct oblimin rotation because the underlying constructs were expected to be correlated. Sampling adequacy was evaluated using the Kaiser–Meyer–Olkin (KMO) measure and Bartlett’s test of sphericity. Items with factor loadings below 0.40 or with cross-loadings differing by less than 0.15 were removed through an iterative process. Each factor was required to contain at least three items. CFA was subsequently conducted using maximum likelihood estimation to examine the factor structure identified through EFA. Model fit was evaluated using multiple fit indices. Convergent validity was assessed using the Average Variance Extracted (AVE) and Construct Reliability (CR), whereas discriminant validity was evaluated by comparing the square root of the AVE for each construct with the inter-factor correlations. Internal consistency reliability was assessed using Cronbach’s α coefficients.

## 3. Results

### 3.1. Participant Characteristics

A total of 500 adolescents participated in this study, including 250 in the exploratory factor analysis (EFA) sample and 250 in the confirmatory factor analysis (CFA) sample. Overall, 147 participants (29.4%) were male and 353 (70.6%) were female. The mean age of the participants was 13.8 years (SD = 0.9). The sample included 234 sixth-grade elementary school students (46.8%), 117 first-year middle school students (23.4%), and 149 second-year middle school students (29.8%). Overall, 59 participants (11.8%) reported previous mental health help-seeking experience. The demographic characteristics of the participants are presented in Table 2.

### 3.2. Item Analysis

Item analysis was conducted for the 33 preliminary items. Corrected item–total correlations ranged from −0.040 to 0.869. Seven items—Items 8 (r = 0.063), 9 (r = 0.066), 10 (r = −0.040), 14 (r = 0.208), 18 (r = 0.260), 27 (r = 0.165), and 30 (r = −0.031)—had corrected item–total correlations below 0.30 and were removed. Consequently, 26 items were retained for EFA.

Table 3 presents the means, standard deviations, ranges, skewness, kurtosis, and corrected item–total correlations for the 26 retained items. Mean item scores ranged from 2.72 to 4.41, and standard deviations ranged from 0.89 to 1.27. Skewness ranged from −1.63 to 0.15, and kurtosis ranged from −0.96 to 2.38, indicating no severe deviations from normality. Corrected item–total correlations for the retained items ranged from 0.314 to 0.869, and all exceeded the criterion of 0.30. Therefore, all 26 items were entered into the subsequent EFA (Table 3).

### 3.3. Exploratory Factor Analysis

Principal axis factoring with direct oblimin rotation was used to identify the underlying factor structure while allowing correlations among the factors. The number of factors was determined by considering the eigenvalues and the inflection point of the scree plot at each stage, together with the factor-loading patterns and theoretical interpretability of the extracted factors. Items with factor loadings below 0.40 were considered for removal. Items for which the difference between the two highest absolute factor loadings was less than 0.15 were considered to have substantial cross-loadings.

Four rounds of EFA were conducted through the sequential removal of items that did not meet the predefined factor-loading or cross-loading criteria. In the first analysis, five factors were specified for the 26 retained items based on inspection of the initial scree plot. Six items (Items 5, 6, 19, 20, 24, and 25) were removed, leaving 20 items. In the second analysis, four factors were specified, and three additional items (Items 7, 28, and 29) were removed, resulting in 17 items. In the third analysis, a three-factor solution was examined, and Item 32 was removed because its factor loading was below 0.40. The fourth and final analysis was conducted using the remaining 16 items, and no additional items met the criteria for removal.

In the final fourth EFA, the Kaiser–Meyer–Olkin value was 0.843, and Bartlett’s test of sphericity was statistically significant (χ^2^ = 1462.99, df = 120, *p* < 0.001), supporting the suitability of the final item set for factor analysis. The final three-factor solution comprised 16 items and explained 46.37% of the total variance (Table 4). Factor loadings ranged from 0.44 to 0.93, and communalities ranged from 0.27 to 0.84. Factor 1, Mental Health Help-Seeking Willingness, comprised nine items, had an initial eigenvalue of 5.15, and explained 28.90% of the variance. Factor 2, Overcoming Stigma in Mental Health Help-Seeking, comprised four items, had an initial eigenvalue of 2.28, and explained an additional 11.47% of the variance. Factor 3, Knowledge of Mental Health Help-Seeking Resources, comprised three items, had an initial eigenvalue of 1.45, and explained an additional 6.00% of the variance.

In the final EFA, the initial eigenvalues for the three retained factors were 5.15, 2.28, and 1.45, respectively, with all three exceeding the conventional criterion of 1.0. The three-factor solution was also supported by the scree plot, the clear factor-loading pattern, and the theoretical interpretability of the factors.

### 3.4. Confirmatory Factor Analysis

**Figure 1:**
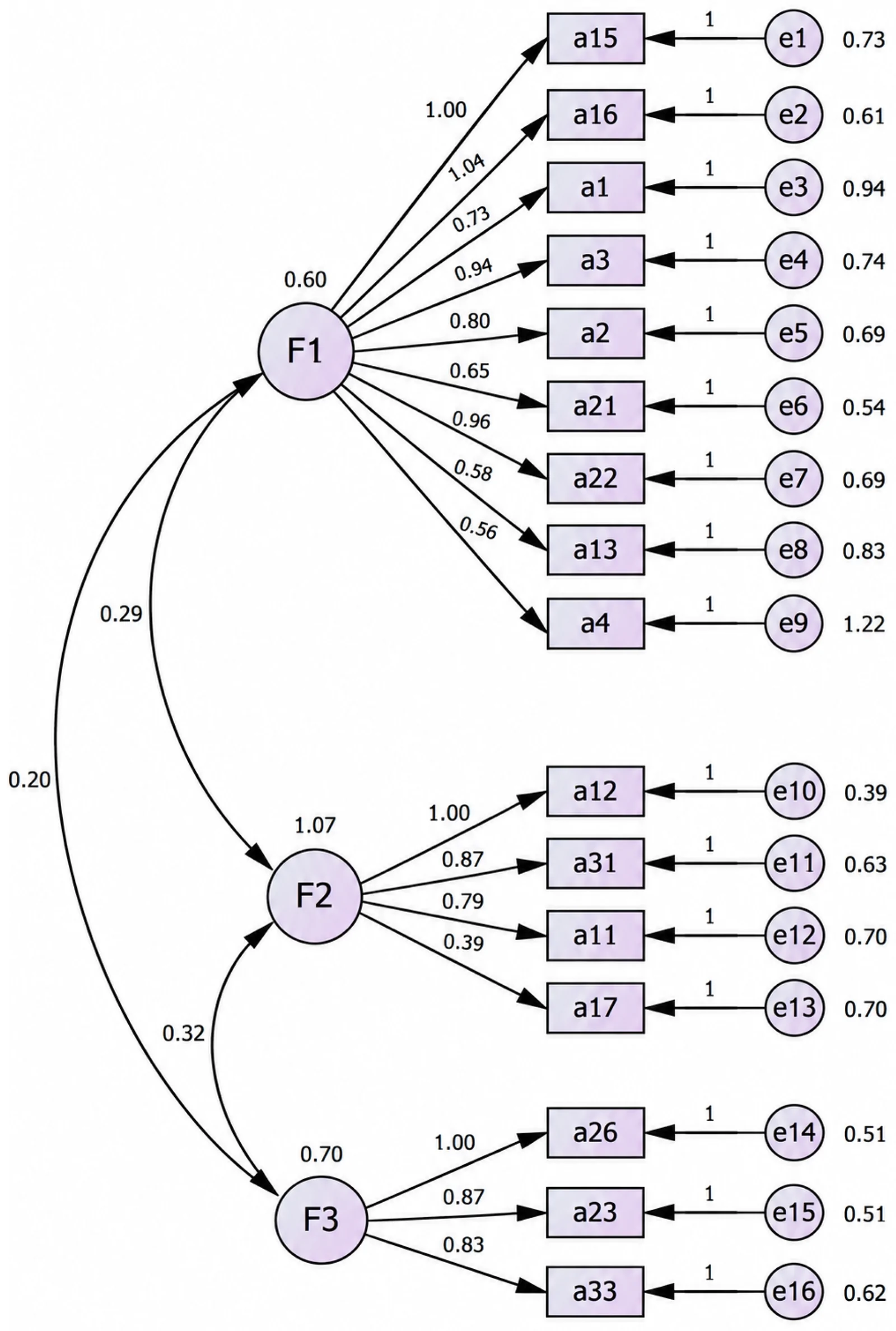
Confirmatory factor analysis of the final three-factor, 16-item Mental Health Help-Seeking Scale. The diagram presents unstandardized parameter estimates. F1 = Mental Health Help-Seeking Willingness; F2 = Overcoming Stigma in Mental Health Help-Seeking; F3 = Knowledge of Mental Health Help-Seeking Resources.

A CFA was conducted to evaluate the three-factor, 16-item model identified through the EFA. Data from the independent CFA sample (n = 250), which did not overlap with the EFA sample, were analyzed using maximum likelihood estimation. The model-fit indices were χ^2^ = 260.22 (df = 101, *p* < 0.001), χ^2^/df = 2.58, GFI = 0.88, AGFI = 0.84, NFI = 0.80, TLI = 0.84, CFI = 0.87, RMR = 0.09, and RMSEA = 0.08 (Table 5). CFA provided preliminary support for the three-factor structure, although several fit indices were below conventional thresholds. Modification indices were reviewed; however, the original model was retained because the suggested modifications were not considered theoretically justified. Figure 1 presents the path diagram of the final model, including the unstandardized factor loadings, error variances, factor variances, and covariances among the latent factors (Table 5).

### 3.5. Convergent and Discriminant Validity

Convergent validity was evaluated using standardized factor loadings, average variance extracted (AVE), and construct reliability (CR). The critical ratio values for all items ranged from 5.21 to 11.26 and exceeded the criterion of 1.96 (*p* < 0.001). The standardized factor loadings ranged from 0.37 to 0.86. The AVE values were 0.34 for Mental Health Help-Seeking Willingness, 0.49 for Overcoming Stigma in Mental Health Help-Seeking, and 0.51 for Knowledge of Mental Health Help-Seeking Resources. The corresponding CR values were 0.82, 0.79, and 0.76, respectively. Evidence for convergent validity was mixed. Although CR exceeded 0.70 for all three factors, the AVE values for Mental Health Help-Seeking Willingness and Overcoming Stigma in Mental Health Help-Seeking were below 0.50, indicating that convergent validity requires further examination. The standardized correlations were 0.36 between Mental Health Help-Seeking Willingness and Overcoming Stigma in Mental Health Help-Seeking, 0.32 between Mental Health Help-Seeking Willingness and Knowledge of Mental Health Help-Seeking Resources, and 0.37 between Overcoming Stigma in Mental Health Help-Seeking and Knowledge of Mental Health Help-Seeking Resources. The squared correlation coefficients ranged from 0.10 to 0.14 and were lower than the AVE values of the corresponding factors, supporting the discriminant validity of the three-factor model (Figure 1; Table 6).

### 3.6. Internal Consistency and Final Scale

The Cronbach’s α coefficient for the overall 16-item Mental Health Help-Seeking Scale was 0.85. The coefficients for the three subscales were 0.85 for Mental Health Help-Seeking Willingness, 0.80 for Overcoming Stigma in Mental Health Help-Seeking, and 0.73 for Knowledge of Mental Health Help-Seeking Resources. These results indicated acceptable internal consistency for the overall scale and all three subscales (Table 6).

The final Adolescent Mental Health Help-Seeking Scale comprises 16 items across three subscales: Mental Health Help-Seeking Willingness, Overcoming Stigma in Mental Health Help-Seeking, and Knowledge of Mental Health Help-Seeking Resources. Items from the same subscale were interspersed to minimize response-pattern bias. Each item is rated on a five-point Likert scale, and the item scores are summed after reverse-scoring the negatively worded items. Given the multidimensional structure of the scale, the three subscale scores should serve as the primary basis for interpretation. A total score may be calculated as a preliminary descriptive indicator, but its interpretation as a general measure of help-seeking readiness requires further empirical validation. The complete scale, including item content and response options, is provided in Supplementary Table S4.

## 4. Discussion

### 4.1. Principal Findings

This study developed and evaluated a 16-item instrument comprising three factors: Mental Health Help-Seeking Willingness, Overcoming Stigma in Mental Health Help-Seeking, and Knowledge of Mental Health Help-Seeking Resources. These factors represent motivational, social, and practical dimensions that may facilitate or impede adolescents’ timely access to appropriate support.

Mental health help-seeking is a multidimensional process involving problem recognition, expression of a need for support, identification of available sources of assistance, and willingness to approach those sources [4]. During adolescence, this process is influenced not only by individual attitudes but also by relationships with peers, parents, teachers, and professionals. Concerns about confidentiality, stigma, limited knowledge of services, negative expectations, and practical access barriers may prevent adolescents from obtaining professional support [7]. Fear of ridicule and negative social evaluation may further discourage them from disclosing mental health concerns [8]. The three factors identified in this study reflect several of these interconnected influences.

A major contribution of the present study is its conceptualization of adolescent mental health help-seeking as a developmentally and culturally situated construct rather than a direct extension of adult help-seeking. Existing instruments, including the ATSPPH and its shortened versions, primarily assess general attitudes toward seeking professional psychological help [9,10,11], while the MHSAS focuses on attitudes toward professional mental health services [12]. Although these instruments provide valuable measures of help-seeking attitudes, they may not fully capture the developmental constraints, peer-related concerns, and practical resource knowledge involved in adolescents’ decisions about seeking support.

The instrument-development process integrated theoretical and fieldwork phases. The theoretical phase was informed by established scale-development and concept-analysis procedures [17,18,19], while the fieldwork phase examined adolescents’ everyday experiences and interpretations of mental health help-seeking. EFA and CFA were subsequently used as complementary methods to identify and evaluate the proposed structure [20,21]. Conducting these analyses with independent samples strengthened the evaluation by separating factor identification from factor confirmation. The resulting three-factor solution explained 46.4% of the total variance and was broadly consistent with the attributes identified through concept analysis and in-depth interviews. However, the proportion of explained variance was relatively modest, indicating that the three-factor solution may not fully capture the complexity of adolescent mental health help-seeking. This finding warrants cautious interpretation and further examination of the factor structure in independent and more diverse adolescent samples.

The fieldwork revealed experiences that may not be adequately represented in adult-oriented instruments. Adolescents expressed concern that being observed entering a school counseling room could lead to rumors or changes in how peers perceived them. Stigma was therefore embedded in the physical and social environment of the school rather than experienced solely as an abstract fear of negative evaluation. The accessibility of mental health support may consequently depend on the visibility of service use, confidentiality, the location of counseling spaces, and the broader social climate surrounding help-seeking [7,8].

Peer reputation may be particularly influential during adolescence because peer relationships and social acceptance are closely connected to social and emotional development [22]. Adolescents who anticipate negative peer reactions may delay seeking assistance even when they recognize a need for support [7,8]. Early-prevention initiatives should therefore address the social context of help-seeking as well as individual knowledge and attitudes.

The interviews also indicated that adolescents did not interpret the abstract term “professional” consistently. Providing specific examples, such as a school counselor, therapist, or psychiatrist, improved comprehensibility and clarified potential sources of support. This finding illustrates the importance of involving the intended population in item development and using language appropriate to adolescents’ developmental level.

In addition, adolescents distinguished between voluntarily requesting support and receiving counseling at the recommendation of a parent, teacher, or other adult. Previous service use alone may therefore not fully reflect recognition of need, willingness to participate, or perceived control over the decision. Help-seeking autonomy may also be constrained by parental consent, financial dependence, transportation, and school schedules; consequently, favorable attitudes may not lead directly to service use when practical access is restricted [7,23]. Low service utilization should therefore not automatically be interpreted as unwillingness. Assessment should also consider limited resource knowledge and structural barriers beyond adolescents’ immediate control.

### 4.2. Interpretation of the Three Factors

The first factor, Mental Health Help-Seeking Willingness, comprised nine items and accounted for the largest proportion of variance. It reflects recognition of a need for support, positive acceptance of professional assistance, and motivation to obtain help when experiencing mental health difficulties. This interpretation is consistent with the conceptualization of help-seeking as a process beginning with the recognition and expression of a need for assistance [4]. It is also consistent with evidence that attitudes, behavioral intentions, perceived control, and autonomy-related barriers influence decisions about seeking professional support [23,24].

Compared with the MHSAS, which primarily focuses on attitudes toward professional psychological help [12], this factor encompasses recognition of the need for assistance, willingness to obtain it, and readiness to recommend help to others. It may therefore capture a broader form of help-seeking readiness among adolescents and help identify those who recognize emotional difficulties but remain uncertain or reluctant to engage with available support [4,12,23,24].

The inclusion of recommending help to others may be particularly relevant because peers are often among the first to notice emotional or behavioral changes. Willingness to recommend support to a friend may reflect positive expectations regarding professional assistance and the normalization of help-seeking within the peer group. However, adolescents should not be expected to manage their peers’ mental health problems. Prevention programs should instead help them recognize concerning signs, respond supportively, and connect peers with trusted adults or qualified professionals.

The second factor, Overcoming Stigma in Mental Health Help-Seeking, comprised four items and reflects willingness to request support despite anticipated social judgment. Mental health stigma among children and adolescents may involve self-stigma, perceived stigma, and experienced stigma, all of which can affect disclosure and service use [25]. Consistent with previous evidence, adolescents in this study expressed concern that peers or family members might regard them as strange, weak, or problematic if they received counseling [7,8,25].

This factor does not merely assess the presence of stigma; it reflects the willingness required to seek assistance despite anticipated negative evaluation. Adolescents may recognize the value of professional support and hold favorable attitudes toward counseling while still avoiding help because they fear that others will become aware of it. However, some conceptual overlap between overcoming stigma and general help-seeking willingness is possible because anticipated stigma may itself influence willingness to seek help. This potential overlap should be examined further in future validation studies.

Because social acceptance and belonging become particularly salient during adolescence, anticipated changes in peer reputation may influence disclosure and the use of school-based services [8,22,25]. Expanding counseling services alone may therefore be insufficient when service use is perceived as socially risky. Stigma-reduction education should be accompanied by efforts to normalize help-seeking, protect confidentiality, strengthen supportive relationships, and establish referral pathways that minimize unwanted exposure.

The third factor, Knowledge of Mental Health Help-Seeking Resources, comprised three items and reflects the ability to identify appropriate sources of support and understand how and where assistance can be obtained. Knowledge of mental health problems and available sources of help is a central component of mental health literacy [26], and help-seeking-related knowledge has been identified as an important area of assessment [27]. Adolescents may recognize that a mental health problem is serious while remaining uncertain about whom to approach, which services are available, or how to initiate contact [7,26,27].

This factor represents more than factual knowledge. It reflects the practical readiness required to translate problem recognition into an approach toward an appropriate source of support. Adolescents may hold favorable attitudes toward professional services but lack information about service locations, referral procedures, parental involvement, confidentiality, or what to expect after initial contact. Assessing this dimension separately may help distinguish insufficient resource knowledge from unwillingness or stigma-related avoidance.

Knowledge of available resources corresponds to the stage in which an individual identifies and approaches an appropriate source of assistance [4], thereby connecting mental health literacy with the practical process of help-seeking [4,26,27]. In educational, clinical, and community settings, low scores may indicate a need for clearer information about available services and procedures for obtaining support. Digital resources may also provide developmentally appropriate guidance, although they should facilitate connections with trusted adults and qualified professionals rather than replace interpersonal or professional care.

Unlike the ATSPPH, its shortened versions, and the MHSAS [9,10,11,12], the present scale encompasses willingness, the capacity to overcome anticipated stigma, and knowledge of available resources. The identification of stigma-related barriers and resource knowledge as potentially distinguishable dimensions is meaningful because favorable attitudes alone may not result in help-seeking when adolescents fear peer judgment or do not know how to access support [7,8,25,26,27].

The three factors may therefore be viewed as conceptually related but potentially distinguishable, although further validation is needed to confirm their empirical distinctiveness. An adolescent may value professional support but avoid it because of anticipated stigma, while another may be willing to seek assistance but lack knowledge of available resources. Given the multidimensional structure identified in this study, the three subscale scores should serve as the primary basis for interpretation, reflecting Mental Health Help-Seeking Willingness, Overcoming Stigma in Mental Health Help-Seeking, and Knowledge of Mental Health Help-Seeking Resources. Although a total score may provide a preliminary descriptive indication of overall help-seeking readiness, its interpretation as a general score requires further empirical validation.

### 4.3. Psychometric Properties and Practical Implications

The CFA provided preliminary support for the proposed three-factor structure. The χ^2^/df ratio and RMSEA were within commonly used acceptable ranges, whereas several incremental fit indices were below conventional thresholds. Modification indices were reviewed, but the original model was retained because the suggested changes were not theoretically justified. This decision preserved the conceptual coherence established through the concept analysis, interviews, and EFA rather than introducing modifications based solely on the characteristics of one sample. Nevertheless, the mixed fit indices indicate that the factor structure should be examined further in independent samples. These findings should also be interpreted in light of the use of maximum likelihood estimation for the five-point ordinal items; therefore, the robustness of the three-factor structure should be further examined using an estimator specifically designed for ordinal data, such as WLSMV. Although the initial eigenvalue for Factor 3 exceeded 1.0 and its retention was supported by the scree plot, factor-loading pattern, and conceptual interpretability, the stability of this three-item factor should still be examined in independent samples.

The overall scale demonstrated satisfactory internal consistency, with a Cronbach’s α of 0.85, and all three subscales demonstrated acceptable reliability. CR exceeded 0.70 for each factor. Evidence for convergent validity was mixed because the AVE values for Mental Health Help-Seeking Willingness and Overcoming Stigma in Mental Health Help-Seeking were below 0.50. In contrast, the low-to-moderate inter-factor correlations and comparison of the squared correlations with the corresponding AVE values supported discriminant validity. The substantial item reduction during psychometric evaluation should also be considered when interpreting the final scale. Although the preliminary item pool was developed to broadly represent the conceptual attributes identified through concept analysis and adolescent interviews and was subsequently subjected to expert content validation, 17 of the 33 preliminary items were removed during empirical evaluation. This indicates that some preliminary items did not perform adequately within the emerging factor structure.

If further validated, the multidimensional structure may support the planning and evaluation of adolescent mental health prevention initiatives across educational, clinical, and community settings. Low Mental Health Help-Seeking Willingness scores may indicate a need to address problem recognition, trust in professional support, and expectations regarding the benefits of early assistance. Difficulty Overcoming Stigma may indicate the need to strengthen supportive social norms, protect confidentiality, and provide referral pathways that minimize unwanted exposure. Limited Knowledge of Mental Health Help-Seeking Resources may suggest a need for clearer information about available services and practical guidance on how to obtain support.

Professionals and organizations working with adolescents may eventually use, following further validation, subscale profiles to identify barriers that could delay help-seeking and to guide developmentally appropriate preventive support. In schools, the scale may inform mental health literacy education, stigma-reduction activities, and referral procedures. Community mental health centers, youth support services, primary care settings, and other adolescent-serving organizations may use the findings to improve service information, strengthen connections with appropriate sources of assistance, and facilitate early access to support.

Following further validation, aggregated subscale scores may help schools and community organizations identify which dimensions could be prioritized when developing or evaluating prevention initiatives. For example, adequate willingness accompanied by limited resource knowledge would indicate a need for clearer information about available services and access procedures. In contrast, adequate knowledge accompanied by difficulty overcoming stigma would suggest that increasing service awareness alone may be insufficient and that the surrounding social environment should also be addressed.

The scale may be useful for evaluating community- and school-based mental health literacy, stigma-reduction, and help-seeking promotion initiatives. It should be interpreted as an assessment of help-seeking-related willingness, stigma-related barriers, and resource knowledge rather than as a diagnostic measure of mental disorders or suicide risk. Its potential value lies in identifying modifiable areas that can be addressed before psychological difficulties escalate or barriers result in prolonged delays in obtaining appropriate support.

### 4.4. Strengths and Limitations

Several strengths of this study should be noted. The instrument-development process integrated concept analysis, in-depth interviews with adolescents, expert content validation, and quantitative psychometric evaluation. This approach enabled the items to reflect both theoretical attributes and the developmental and sociocultural experiences of Korean adolescents. The use of language and examples that adolescents could readily understand further strengthened the developmental relevance of the items. In addition, conducting EFA and CFA with independent samples reduced the likelihood that the identified structure reflected characteristics specific to a single analytic sample.

The findings should nevertheless be interpreted in light of the sampling characteristics. Participants were recruited from elementary and middle schools in one region of Korea, limiting generalizability to adolescents from other geographic, cultural, and educational settings. Female adolescents were overrepresented, and the sample was limited to those aged 11–14 years, primarily representing early adolescence. Therefore, the findings should not be generalized to the broader adolescent population, particularly older adolescents, without further validation. Further studies should evaluate the scale in geographically diverse samples, with more balanced gender distributions, and across a broader age range encompassing both early and later adolescence.

Several measurement properties also require further investigation. Test–retest reliability, criterion-related validity, predictive validity, and measurement invariance across gender and age groups were not evaluated. Furthermore, the present study did not evaluate a higher-order or general-factor model; therefore, the empirical basis for interpreting the 16 items as a single overall score remains limited. Further studies should examine whether a general score is psychometrically supported in addition to the three subscale scores. Moreover, CFA was conducted using maximum likelihood estimation despite the ordinal nature of the five-point Likert items. This should be considered a methodological limitation when interpreting the present CFA findings. Future studies should examine the factor structure using estimators specifically designed for ordinal data, such as WLSMV based on polychoric correlations, to evaluate the robustness of the proposed factor structure. In addition, direct quantitative comparisons with established help-seeking measures, such as the ATSPPH and MHSAS, were not conducted because these measures were not administered concurrently. Future studies should examine whether the factor structure is replicated in independent samples, whether scores remain stable over time and whether scores are associated with established help-seeking measures and actual help-seeking behavior. Further cross-cultural evaluation would also clarify the applicability of the scale beyond the population examined in this study.

Despite these limitations, the integration of theoretical, qualitative, and quantitative development procedures provides a relevant foundation for assessing mental health help-seeking among Korean adolescents. The scale may be useful for identifying help-seeking willingness, stigma-related barriers, and knowledge of available support resources and for planning and evaluating developmentally and culturally responsive adolescent mental health prevention initiatives across school, clinical, and community settings.

## 5. Conclusions

This study developed a 16-item scale comprising three dimensions of mental health help-seeking among Korean adolescents: Mental Health Help-Seeking Willingness, Overcoming Stigma in Mental Health Help-Seeking, and Knowledge of Mental Health Help-Seeking Resources. The findings provide preliminary support for the scale’s factor structure, reliability, and construct validity. The scale may provide a basis for assessing help-seeking-related willingness, stigma-related barriers, and knowledge of available support resources among adolescents. However, the proposed factor structure should be considered preliminary and requires further validation. Further studies should examine its psychometric properties across diverse regions, age groups, gender distributions, and cultural contexts and assess its relationships with actual help-seeking behavior.

## Figures and Tables

**Table 1:** Integration of Findings from Concept Analysis and In-Depth Interviews on Adolescent Mental Health Help-Seeking.

Theoretical Attributes Identified Through Concept Analysis	Fieldwork Attributes Identified Through In-Depth Interviews	Final Integrated Attributes
Fear of negative evaluation and stigma	Fear of social stigma and negative evaluation	Fear of social stigma
Difficulty recognizing mental health problems	Recognizing emotional crises	Recognition of emotional crises
Difficulties in help-seeking due to passive coping	Wanting help but being unable to decide alone	Positive and proactive attitude toward help-seeking
Seeking help only within trusted relationships	Searching for available sources of help	Knowledge of help-seeking resources
Help-seeking requires personal determination	Making the decision to seek help	Courage to seek help

Note. Final integrated attributes were synthesized by integrating findings from the theoretical phase (concept analysis) and the fieldwork phase (in-depth interviews with adolescents). These attributes served as the conceptual foundation for developing the initial item pool.

**Table 2:** Characteristics of the Study Participants.

Characteristic	Category	Total (N = 500) n (%)	EFA (N = 250) n (%)	CFA (N = 250) n (%)
Sex	Male	147 (29.4)	71 (28.4)	76 (30.4)
	Female	353 (70.6)	179 (71.6)	174 (69.6)
Age (years)	Mean ± SD	13.8 ± 0.9	13.8 ± 0.9	13.8 ± 0.9
School grade	Grade 6 (elementary school)	234 (46.8)	117 (46.8)	117 (46.8)
	Grade 7 (middle school)	117 (23.4)	59 (23.6)	58 (23.2)
	Grade 8 (middle school)	149 (29.8)	74 (29.6)	75 (30.0)
Previous mental health help-seeking experience	Yes	59 (11.8)	26 (10.4)	33 (13.2)
	No	441 (88.2)	224 (89.6)	217 (86.8)

Note. Values are presented as *n* (%) unless otherwise indicated. EFA = exploratory factor analysis; CFA = confirmatory factor analysis; SD = standard deviation.

**Table 3:** Descriptive Statistics and Item Analysis of the 26 Items Retained for Exploratory Factor Analysis.

Item	M	SD	Minimum	Maximum	Skewness	Kurtosis	Corrected Item–Total Correlation
1	3.36	1.1	1	5	−0.47	−0.52	0.444
2	3.64	1.0	1	5	−0.57	−0.11	0.593
3	3.39	1.12	1	5	−0.29	−0.57	0.642
4	3.38	1.18	1	5	−0.21	−0.96	0.477
5	3.73	1.09	1	5	−0.64	−0.33	0.566
6	3.56	1.27	1	5	−0.52	−0.91	0.487
7	2.72	1.1	1	5	0.15	−0.78	0.402
11	4.11	1.19	1	5	−1.23	0.45	0.499
12	3.85	1.2	1	5	−0.73	−0.4	0.376
13	3.02	1.03	1	5	−0.18	0.02	0.427
15	3.15	1.12	1	5	−0.22	−0.75	0.523
16	3.11	1.12	1	5	−0.24	−0.65	0.576
17	4.41	0.9	1	5	−1.63	2.38	0.337
19	3.78	0.95	1	5	−0.57	0.09	0.363
20	3.35	1.0	1	5	−0.16	−0.63	0.338
21	3.53	0.9	1	5	−0.5	0.18	0.567
22	3.36	1.11	1	5	−0.36	−0.63	0.551
23	3.35	1.02	1	5	−0.35	−0.1	0.329
24	3.9	0.89	1	5	−0.89	1.38	0.454
25	3.37	1.15	1	5	−0.43	−0.65	0.507
26	3.71	1.1	1	5	−0.64	−0.22	0.564
28	3.94	0.93	1	5	−0.85	0.96	0.371
29	3.81	1.05	1	5	−0.72	−0.09	0.535
31	3.66	1.18	1	5	−0.56	−0.69	0.314
32	3.43	1.11	1	5	−0.37	−0.44	0.562
33	3.72	1.01	1	5	−0.58	−0.07	0.869

Note. M = mean; SD = standard deviation.

**Table 4:** Results of the Exploratory Factor Analysis of the Mental Health Help-Seeking Scale (N = 250).

No.	Factor 1	Factor 2	Factor 3	Communality
15	0.74			0.49
16	0.73			0.52
1	0.68			0.42
3	0.65			0.50
2	0.64			0.48
21	0.60			0.41
22	0.56			0.39
13	0.51			0.27
4 *	0.44			0.27
12 *		0.93		0.84
31 *		0.75		0.52
11 *		0.67		0.54
17 *		0.48		0.30
26			0.72	0.66
23			0.62	0.36
33			0.60	0.46
Initial eigenvalue	5.15	2.28	1.45	
Variance explained (%)	28.90	11.47	6.00	
Cumulative variance (%)	28.90	40.37	46.37	

Note. Principal axis factoring with direct oblimin rotation was used. KMO = 0.843; Bartlett’s test of sphericity: χ^2^ = 1462.99, df = 120, *p* < 0.001. * Reverse-coded items. Factor 1 represents Mental Health Help-Seeking Willingness; Factor 2 represents Overcoming Stigma in Mental Health Help-Seeking; and Factor 3 represents Knowledge of Mental Health Help-Seeking Resources.

**Table 5:** Goodness-of-Fit Indices of the Final Three-Factor Model (N = 250).

χ^2^	df	χ^2^/df	GFI	AGFI	NFI	TLI	CFI	RMR	RMSEA
260.22	101	2.58	0.88	0.84	0.80	0.84	0.87	0.09	0.08

Note. χ^2^ = chi-square statistic; df = degrees of freedom; GFI = goodness-of-fit index; AGFI = adjusted goodness-of-fit index; NFI = normed fit index; TLI = Tucker–Lewis index; CFI = comparative fit index; RMR = root mean square residual; RMSEA = root mean square error of approximation.

**Table 6:** Construct Validity and Internal Consistency of the Final Mental Health Help-Seeking Scale (N = 250).

Dimension	Items	AVE	CR	Cronbach’s α	
Mental Health Help-Seeking Willingness	9	0.34	0.82	0.85	0.85
Overcoming Stigma in Mental Health Help-Seeking	4	0.49	0.79	0.80	
Knowledge of Mental Health Help-Seeking Resources	3	0.51	0.76	0.73	

Note. AVE = average variance extracted; CR = construct reliability.

## Data Availability

The data presented in this study are available on request from the corresponding author. The data are not publicly available due to privacy and ethical considerations related to research involving adolescent participants.

## Supplementary Materials

The following supporting information can be downloaded at: https://www.mdpi.com/article/10.3390/children13081108/s1, Supplementary Table S1. Assessment of Reporting Completeness Using the COSMIN Reporting Checklist; Supplementary Table S2. Initial Item Pool of the Adolescent Mental Health Help-Seeking Scale (50 Items); Supplementary Table S3. Preliminary 33-Item Version of the Adolescent Mental Health Help-Seeking Scale Following Content Validation; Supplementary Table S4. Mental Health Help-Seeking Scale for Adolescents.
