# Supplementary material for: Development and Psychometric Evaluation of a Mental Health Help-Seeking Scale for Korean Adolescents
Source: Children (Basel). 2026 Aug 19;13(8):1108. doi: 10.3390/children13081108 (PMC13511209; doi:10.3390/children13081108)
Supplement: Supplementary file 1 [file children-13-01108-s001.zip › children-4493624-supplementary.pdf]

Supplementary Table S1. COSMIN-Informed Assessment of Reporting Completeness

No.	Item	Reported
**1**	The title identifies the study as instrument development and/or validation research.	Title page
**2**	The abstract clearly states the study objective.	Page 2
**3**	The abstract describes the target population.	Page 2
**4**	The abstract summarizes the measurement properties evaluated.	Page 2
**5**	The construct to be measured is clearly defined.	Pages 3 and 5–6
**6**	The rationale for developing the instrument is described.	Pages 3–4
**7**	Existing instruments and their limitations are discussed.	Page 4
**8**	The study design is clearly described.	Page 5
**9**	Eligibility criteria for participants are reported.	Page 9

**10**	Sampling procedures are described.	Pages 6 and 10
**11**	Sample size justification is provided.	Page 10
**12**	Participant characteristics are reported.	Pages 12–13; Table 2
**13**	A conceptual framework for the construct is described.	Pages 5–7; Table 1
**14**	Procedures used for item generation are reported.	Pages 7–8
**15**	Sources used to generate items are described.	Pages 6–8
**16**	Expert review procedures are reported.	Pages 7–8
**17**	Characteristics and number of experts are described.	Page 8
**18**	Methods used to assess content validity are reported.	Page 8
**19**	Pilot testing or cognitive interviews are described.	Page 9
**20**	Statistical methods used for exploratory factor analysis are reported.	Pages 11 and 15

**21**	Factor extraction methods are described.	Pages 11 and 15
**22**	Rotation methods are reported.	Pages 11 and 15
**23**	Criteria used to determine the number of factors are described.	Pages 11 and 15–16
**24**	Factor loadings are presented.	Pages 16–17; Table 4
**25**	Explained variance and/or eigenvalues are reported.	Pages 16–17; Table 4
**26**	Confirmatory factor analysis procedures are reported, if applicable.	Pages 11 and 18
**27**	Model fit indices are reported and interpreted.	Page 18; Table 5

**28**	Internal consistency statistics (e.g., Cronbach's α) are reported.	Pages 19–20 and 29; Table 6
**29**	Construct validity testing (e.g., convergent/discriminant validity) is reported.	Pages 18–19 and 29; Table 6
**30**	Study limitations are discussed.	Pages 31–32
**31**	Implications for practice, research, or future instrument use are described.	Pages 29–33

Supplementary Table S2. Initial Item Pool of the Adolescent Mental Health Help-Seeking Scale (50 Items)

No.	Item
1	If I felt anxious, emotionally distressed, and overwhelmed, I would want someone capable of providing professional help to reach out to me.
2	When I feel emotionally distressed, I believe that counseling with a professional could help me regain emotional stability.
3	If I became so emotionally distressed that I could no longer endure it, I would make an effort to seek help from a mental health professional.
4	I believe that I could experience a mental health problem at any point in my life and that I might seek counseling on my own.
5	Because I believe that people should solve their own problems, receiving counseling would be my last option.
6	Even if I am emotionally distressed now, the problem will resolve itself if I do not talk about it and simply leave it alone.
7	I admire people who try to overcome their concerns or difficulties on their own without receiving professional help, such as counseling.
8	If I received counseling, I would doubt whether it would actually help me and might feel that it was a waste of time and money.
9	I do not think that sharing my problems with a counselor and discussing them together is a good way to relieve my emotional distress.
10	If I experienced emotional difficulties, I would probably talk to a family member, teacher, or another adult.
11	If I experienced emotional difficulties, I would probably talk to a friend.
12	I believe that receiving professional help because my parents told me to is different from voluntarily seeking counseling because I wanted it.
13	To receive professional help, I should seek it on my own without discussing it with anyone else.
14	If I received professional help for my mental health, my family or other people would think that I was strange.
15	If I received professional help for my mental health, my friends would think that I was strange.
16	I would like to recommend that my friends receive professional help for their mental health.
17	Strong people can overcome emotional distress on their own and do not need professional help.
18	If I felt so emotionally distressed that I seemed completely lost, I would want to hear

	a professional's opinion about what I was experiencing.
19	If I frequently became angry for no apparent reason and could not stop worrying, I would want to receive professional help for my mental health.
20	I admire people who find ways to resolve their emotional problems on their own.
21	I am afraid that other people might find out that I am receiving counseling for my mental health.
22	Having a mental health problem is something to be ashamed of.
23	I would not want to hide from others that I was receiving counseling for my mental health.
24	If I believed that I needed professional help for my mental health, I would begin counseling regardless of whether other people found out.
25	If I believed that it would benefit my mental health, I would disclose my concerns to a professional even if they involved personal or confidential matters.
26	Some problems should not be discussed with anyone other than close family members.
27	To stop worrying, it is better to distract myself by focusing on something else.
28	Even if a professional counselor could help me, I would strongly dislike the counselor knowing about my personal difficulties and confidential matters.
29	I have personal experiences that I would not want to discuss with anyone.
30	Even if someone is very close to me, I would not want that person to know everything about me.
31	Although hospitals provide services for people with mental health problems, I do not think that such services would be very helpful for my mental health.
32	If a close friend were concerned about a mental health problem, I would recommend that the friend meet with a counselor who could provide professional help.
33	I believe that it is better to endure a mental health problem than to receive counseling to resolve it.
34	I believe that a person with a serious mental health problem could feel more at ease in an appropriate mental health treatment setting.
35	Even for a serious problem, I would rather listen to the advice of a close friend than seek a professional opinion.
36	Because it is difficult for people with mental health problems to resolve those problems on their own, they should receive professional help.
37	If I experienced a serious problem related to school violence, I would know what to do to address it.
38	If a friend experienced a serious problem related to school violence, I would know what to do to help address it.
39	If thoughts about playing games occupied my mind all day and prevented me from

	concentrating on daily life, I would know what to do to address the problem.
40	If I encountered a friend who was thinking about suicide, I would know what to do.
41	Some problems cannot be resolved by telling friends and require asking an adult for help.
42	If I were assured that what I shared would remain completely confidential, I would feel able to talk openly about my concerns.
43	I can recognize when I am emotionally distressed.
44	If counseling successfully resolved a mental health problem, I would seek counseling again if I experienced a similar problem in the future.
45	I would need to experience counseling before I could determine whether it would be helpful to me.
46	If other students saw me entering the counseling office, they would think that I was strange.
47	I know how to seek help when I am emotionally distressed.
48	Receiving help from others for my mental health is a way of protecting myself.
49	If my first counseling experience remained a positive memory, I would be willing to receive counseling again when facing future difficulties.
50	There are times when my emotional distress becomes too difficult to endure and I feel that I need to talk to someone to feel better.

Note. The 50 items presented in this table constitute the initial item pool generated through concept analysis and in-depth interviews. The English translations are provided for informational purposes and have not undergone independent linguistic or psychometric validation. These items should not be administered as the final scale. The validated Korean scale consists of 16 items.

Supplementary Table S3. Preliminary 33-Item Version of the Adolescent Mental Health Help-Seeking Scale Following Content Validation

No.	Item
1	If I felt anxious and emotionally distressed, I would want someone capable of providing professional help (e.g., counseling or treatment) to reach out to me.
2	When I feel emotionally distressed, I believe that help from a professional (e.g., a school counselor or therapist) could help me feel more at ease.
3	If I became so emotionally distressed that I could no longer endure it, I would make an effort to seek help from a mental health professional (e.g., a school counselor or therapist).
4*	When I feel emotionally distressed, the problem will improve on its own if I simply leave it alone.
5*	I do not think that sharing my concerns with a professional (e.g., a school counselor or therapist) is a good way to relieve my emotional distress.
6	If I experienced emotional difficulties, I would probably talk to a family member first.
7	If I experienced emotional difficulties, I would probably talk first to a teacher or an adult whom I trust.
8	If I experienced emotional difficulties, I would probably talk to a friend first.
9	When I am emotionally distressed, I believe that being made to receive professional help (e.g., treatment or counseling) at the suggestion of someone else, such as a parent or teacher, is different from voluntarily seeking such help on my own.
10*	To receive professional help for my mental health (e.g., treatment or counseling), I only need to seek a professional (e.g., a school counselor or therapist) on my own without discussing it with anyone else.
11*	If I decided to receive professional help for my mental health (e.g., treatment or counseling), my family would think that I was strange.
12*	If I decided to receive professional help for my mental health (e.g., treatment or counseling), my friends would think that I was strange.
13	I would like to recommend that my friends receive professional help for their mental health (e.g., treatment or counseling).
14*	Strong people can overcome emotional difficulties on their own, and only weak people need professional help (e.g., treatment or counseling).
15	If I suddenly felt emotionally distressed and hopeless for no apparent reason, I would want to hear a professional's opinion (e.g., a school counselor's or therapist's) about why I was feeling this way.
16	If I became increasingly angry for no apparent reason and could not stop worrying, I

	would want to receive professional help for my mental health (e.g., treatment or counseling).
17*	Having a mental health problem is something to be ashamed of.
18	I know how to find information that can help improve my mental health.
19	Mental health education (e.g., suicide prevention, school violence prevention, or drug addiction prevention) teaches me how to obtain help from others.
20*	Even for a serious mental health problem, I would rather listen to the advice of a close friend than seek the opinion of a professional (e.g., a school counselor or therapist).
21	Because it is difficult for people with mental health problems to resolve those problems on their own, they should receive professional help.
22	If it would benefit my mental health, I believe that I should disclose my concerns to a professional (e.g., a school counselor or therapist), even if they involve personal or confidential matters.
23	If I experienced a serious mental health problem, I would know how to seek help to address it.
24	Receiving help for my mental health is a way of protecting myself from potentially harmful situations.
25	If thoughts about the same thing, such as games or videos, occupied my mind all day and prevented me from concentrating on daily life, I would feel that I should seek help from a professional (e.g., a school counselor or therapist).
26	If I felt emotionally distressed and no longer wanted to live, I would know how to ask people around me for help.
27	Some mental health problems cannot be resolved by talking to friends and require help from an adult.
28	I can recognize when I am becoming increasingly emotionally distressed.
29	If a mental health problem was successfully resolved with help from a professional (e.g., a school counselor or therapist), I would want to seek professional help again if I experienced a similar problem in the future.
30*	I would need to meet with a professional (e.g., a school counselor or therapist) before I could determine whether professional help would be useful in resolving a mental health problem.
31*	If other students happened to see me meeting with a mental health professional (e.g., a school counselor or therapist), they would think that I was strange.
32	If I needed professional help for my mental health (e.g., counseling or treatment), I would seek it regardless of whether other people found out.
33	If I experienced a serious problem in a friendship that I could not resolve on my own and felt completely overwhelmed, I would know how to ask people around me for

	help.

Note. The 33 items presented in this table constitute the preliminary item pool retained following expert content-validity evaluation. Items 4, 5, 10, 11, 12, 14, 17, 20, 30, and 31 are reverse-coded. The English translations are provided for informational purposes and have not undergone independent linguistic or psychometric validation. These items should not be administered as the final scale; the validated Korean scale consists of 16 items.

Supplementary Table S4. Mental Health Help-Seeking Scale for Adolescents

<Adolescent Mental Health Help-Seeking Scale>

- **The following items concern mental health help-seeking among adolescents.**
- **Please read each item and select the response that best describes you.**
- **Response options**

  1 = Strongly disagree 2 = Disagree 3 = Neutral 4 = Agree

  5 = Strongly agree

Factor	No.	Item	Response options				
Mental health help-seeking willingness	1	If I felt anxious and emotionally distressed, I would want someone capable of providing professional help (e.g., counseling or treatment) to reach out to me.	①	②	③	④	⑤
	2	If I became increasingly angry for no apparent reason and could not stop worrying, I would want to receive professional help for my mental health (e.g., treatment or counseling).	①	②	③	④	⑤
	3	When I feel emotionally distressed, I believe that help from a professional (e.g., a school counselor or therapist) could help me feel more at ease.	①	②	③	④	⑤
	4	Because it is difficult for people with mental health problems to resolve those problems on their own, they should receive professional help.	①	②	③	④	⑤
	5	If I became so emotionally distressed	①	②	③	④	⑤

		that I could no longer endure it, I would make an effort to seek help from a mental health professional (e.g., a school counselor or therapist).					
	6	If it would benefit my mental health, I believe that I should disclose my concerns to a professional (e.g., a school counselor or therapist), even if they involve personal or confidential matters.	①	②	③	④	⑤
	7	When I feel emotionally distressed, the problem will improve on its own if I simply leave it alone.	①	②	③	④	⑤
	8	I would like to recommend that my friends receive professional help for their mental health (e.g., treatment or counseling).	①	②	③	④	⑤
	9	If I suddenly felt emotionally distressed and hopeless for no apparent reason, I would want to hear a professional's opinion (e.g., a school counselor's or therapist's) about why I was feeling this way.	①	②	③	④	⑤
Overcoming stigma in mental health help-seeking	10	If I decided to receive professional help for my mental health (e.g., treatment or counseling), my family would think that I was strange.	①	②	③	④	⑤
	11	If I decided to receive professional help	①	②	③	④	⑤

		for my mental health (e.g., treatment or counseling), my friends would think that I was strange.					
	12	Having a mental health problem is something to be ashamed of.	①	②	③	④	⑤
	13	If other students happened to see me meeting with a mental health professional (e.g., a school counselor or therapist), they would think that I was strange.	①	②	③	④	⑤
Knowledge of mental health help-seeking resources	14	If I experienced a serious mental health problem, I would know how to seek help to address it.	①	②	③	④	⑤
	15	If I felt emotionally distressed and no longer wanted to live, I would know how to ask people around me for help.	①	②	③	④	⑤
	16	If I experienced a serious problem in a friendship that I could not resolve on my own and felt completely overwhelmed, I would know how to ask people around me for help.	①	②	③	④	⑤
